# The effect of area deprivation on COVID-19 risk in Louisiana

**DOI:** 10.1371/journal.pone.0243028

**Published:** 2020-12-03

**Authors:** Madhav K. C., Evrim Oral, Susanne Straif-Bourgeois, Ariane L. Rung, Edward S. Peters

**Affiliations:** 1 Epidemiology Program, School of Public Health, Louisiana State University Health Sciences Center New Orleans, New Orleans, LA, United States of America; 2 Biostatistics Program, School of Public Health, Louisiana State University Health Sciences Center New Orleans, New Orleans, LA, United States of America; Osakidetza Basque Health Service, SPAIN

## Abstract

**Background:**

Louisiana in the summer of 2020 had the highest per capita case count for COVID-19 in the United States and COVID-19 deaths disproportionately affects the African American population. Neighborhood deprivation has been observed to be associated with poorer health outcomes. The purpose of this study was to examine the relationship between neighborhood deprivation and COVID-19 in Louisiana.

**Methods:**

The Area Deprivation Index (ADI) was calculated and used to classify neighborhood deprivation at the census tract level. A total of 17 US census variables were used to calculate the ADI for each of the 1148 census tracts in Louisiana. The data were extracted from the American Community Survey (ACS) 2018. The neighborhoods were categorized into quintiles as well as low and high deprivation. The publicly available COVID-19 cumulative case counts by census tract were obtained from the Louisiana Department of Health website on July 31, 2020. Descriptive and Poisson regression analyses were performed.

**Results:**

Neighborhoods in Louisiana were substantially different with respect to deprivation. The ADI ranged from 136.00 for the most deprived neighborhood and –33.87 in the least deprived neighborhood. We observed that individuals residing in the most deprived neighborhoods had almost a 40% higher risk of COVID-19 compared to those residing in the least deprived neighborhoods.

**Conclusion:**

While the majority of previous studies were focused on very limited socio-environmental factors such as crowding and income, this study used a composite area-based deprivation index to examine the role of neighborhood environment on COVID-19. We observed a positive relationship between neighborhood deprivation and COVID-19 risk in Louisiana. The study findings can be utilized to promote public health preventions measures besides social distancing, wearing a mask while in public and frequent handwashing in vulnerable neighborhoods with greater deprivation.

## Introduction

On March 09, 2020, Louisiana reported its first case of COVID-19 and soon thereafter appeared to be a hot spot of the coronavirus pandemic in the US [1]. Within two weeks of the initial confirmed case, the state had one of the world’s highest average daily growth rate [2–4]. As of July 31, 2020, the state of Louisiana had the highest per capita case count in the United States with a total of 116,280 confirmed cases and 3,835 deaths [2]. The incidence and mortality rates of COVID-19 have been disproportionate across racial and ethnic groups [5, 6]. Specifically, non-Hispanic African Americans have higher rates of incidence, hospitalization, and death from COVID-19 compared to non-Hispanic Whites. In early July, the US Centers for Disease Control and Prevention (CDC) estimated that non-Hispanic African Americans have 4.7 times the rate of age-adjusted COVID-19 related hospitalization rates than non-Hispanic Whites [7]. The sources of disparities in COVID-19 outcomes might be explained from a social determinants of health perspective. Non-Hispanic African Americans are more likely to have vulnerable and low-paying jobs that do not allow remote work, which increases risk of contracting COVID-19 [8, 9]. Furthermore, non-Hispanic African Americans are more likely to rely on public transportation and to live in crowded housing or work in crowded worksite that places them an increased risk for COVID-19 disease. African Americans exhibit a greater burden of chronic medical conditions, such as hypertension, diabetes, heart disease, chronic disease, and obesity that increase the severity of COVID-19 illness [10–12]. In Louisiana, 2.9 million people have at least one chronic condition, and a total of 68 percentage of Louisiana adults are overweight or obese [13]. Furthermore, the poverty rate is much higher among African Americans compared to non-Hispanic Whites, and African Americans tend to live in neighborhoods with high poverty [14, 15]. Neighborhood socioeconomic status (SES) is linked to access to health care services, with people residing in low SES neighborhoods being less likely to have access to health care services, which further increases the risk of adverse health outcomes related to COVID-19, such as higher hospitalizations and mortality [16, 17].

Preliminary reports show a relationship between the neighborhood of residence and COVID-19 disease, hospitalization and death [18–20]. Those who reside in deprived neighborhoods, defined by low income and education, higher unemployment, and substandard living conditions, have a greater risk of poor health outcomes such as obesity, diabetes, cancer, and heart diseases [21–23]. Higher incidence and mortality from COVID-19 have also been observed in low-income or deprived neighborhoods [24–26]. A study conducted by Bilal et al. reported a 36% higher incidence of COVID-19 infection in deprived neighborhoods compared to less deprived neighborhoods [25]. Systemic health, social, and income inequities are considered as the primary reasons that have contributed to the increased risk of contracting COVID-19 in persons residing in deprived neighborhoods [7, 27, 28].

Risk factors leading to COVID-19 disease, hospitalization, and mortality exist not only at the individual or biological level; neighborhood-level factors and their interactions with individual-level factors are also responsible for the observed disparities. Lack of access to health care, unemployment, less education, and poor housing conditions significantly increase the risk of COVID-19 infection [28–31]. These determinants of health can be studied collectively as neighborhood or area deprivation.

Socioeconomic characteristics of residential neighborhoods influence health-related behaviors, conditions, and health outcomes [32, 33]. Deprived neighborhoods are correlated with health risk behaviors, overcrowding, less social cohesion, and higher levels of environmental pollutants and have been identified as critical social determinants of health [34–37]. Low socioeconomic status (SES), often regarded as a fundamental cause of disease, has been shown to increase the risk of COVID-19 because it impacts access to fundamental resources that an individual or a neighborhood may require to avoid COVID-19 [24, 38].

Neighborhoods with a higher number of people per household or room tend to have a higher rate of confirmed COVID-19 cases than neighborhoods with fewer residents [25, 39, 40]. Individuals who share a room or live in overcrowded housing and the use of public transportation often spread the disease rapidly as distancing preventive measures are impossible to adopt.

The primary purpose of this paper is to investigate the relationship between neighborhood deprivation and COVID-19 risk in Louisiana. We hypothesize that deprived Louisiana neighborhoods have a higher risk of COVID-19 reported cases than less deprived neighborhoods, as measured by the Area Deprivation Index (ADI). The ADI is a composite measure of neighborhood socioeconomic disadvantage, created by Gopal K Singh in 2003 [41]. The ADI, composed of 17 education, employment, housing-quality, and poverty census derived measures, is a robust metric measuring many relevant social determinants of health that may help explain the socio-biologic mechanisms of disease [41, 42]. To date, few studies in the US and none in Louisiana have assessed the role of social determinants of health on COVID-19 disease. The studies that exist are limited, examining only a couple of specific risk factors, such as overcrowding and income.

## Materials and methods

### Study data

Publicly available data on cumulative COVID-19 cases by census tract was obtained from the Louisiana Department of Health website on July 31, 2020 [2]. There are 64 parishes (counties) and 1,148 census tracts in Louisiana. The median population size of the census tracts was 4,138. The lowest number of people living in a census tract was 555 and the highest was 18,524. All 64 parishes have reported cases of COVID-19. Because the census tract is considered a good proxy for neighborhood, census tract was selected as the unit of analysis for this study [43]. We extracted the American Community Survey (ACS) 2018 data for census tract level measures for Louisiana [44].

#### COVID-19

The main outcome in this study was cumulative COVID-19 tested positive cases per 1,000 persons in Louisiana census tracts as of July 31, 2020.

#### Neighborhood deprivation

Neighborhood deprivation was measured by the ADI, as described by Singh in 2003 [41]. ADI is a validated, factor-based deprivation index that uses 17 census derived measures of poverty, education, housing, and employment indicators at the census tract level to classify the neighborhoods [41, 45]. More deprived or disadvantaged neighborhoods are those with a higher ADI score. The Quintile ranks were calculated based on ADI scores. Each census tract was assigned an ADI score, and then sorted, and ranked by that score. The first quintile included the 20% of census tracts with the lowest ADI scores and the fifth quintile included the 20% of census tracts with the highest ADI scores, and so forth for the other quintiles.

The census derived indicators used in the calculation of ADI include educational distribution (percentage of the population with less than 9 years and with 12 or more years of education), median family income, median home value, median gross rent, median monthly mortgage, income disparity, unemployment, percent employed person in white-collar occupation, percent families below poverty, percent population below 150% poverty threshold, single-parent household rate, homeownership rate, percent household without a telephone, percent household without a motor vehicle, percent occupied housing units without complete plumbing, and household crowding [41, 45].

*Calculation of ADI score*. Data from the Census Bureau’s American Community Survey (ACS) 2018 were used to calculate the census tract ADI score. The 2018 ADI scores we used for Louisiana census tracts were based upon factor score coefficients initially calculated by Singh [41]. These score coefficients were estimated using a factor analysis of national data to identify the indicators. Out of an initial 21 indicators identified by Singh, 17 indicators had the largest loadings on the first factor. The first factor showed an empirically meaningful clustering of the indicators and have been subsequently validated as the indicators retained in the ADI. The 17 US census indicators were multiplied by the Singh’s coefficients (factor weights) for all census tracts in Louisiana [41, 46]. The base score of each indicator was summed to get the total base score for a census tract. Each census tract’s base score was standardized by dividing the difference between the individual census tract base score (*b*) and the Louisiana census tract population mean (*p*), by Louisiana census tract population standard deviation (*S_p_*) [46].

$${Standard\;base}_{j}=\frac{b-p}{S_{p}},\;j=1,2,\ldots .k,$$

where *j* represents the *j*^th^ census tract, and *k* is the total number of census tracts in Louisiana. Finally, the standardized values were adjusted to a base mean of 100 and a standard deviation of 20 as suggested by Knighton et al. [46].

$$ADI_{j}=(Standard\;base_{j}+100)*20.$$

The details of ADI calculation and a list of variables included in the calculations can be found in Knighton et al. [46].

A total of 31 non-residential, predominately rural census tracts did not have data for the ADI components and were thus excluded from the analysis. The final analytical sample size included 1,127 census tracts.

Census tracts in Louisiana were categorized into rural and urban based on the rural-urban commuting area (RUCA) codes [47]. Census tracts with a RUCA code less than 3 were categorized as urban, and census tracts with a RUCA code greater than 3 were categorized as rural. The final model was adjusted for this rural-urban indicator.

### Statistical analysis

SAS 9.4 software was used for statistical analyses. Heat maps were created using ArcGIS software. Mean, standard deviation, median and interquartile range (IQR) of ADI census indicators by quintile (least deprived: Q1 and most deprived: Q5) were calculated for all census tracts in Louisiana. Poisson regression was performed to estimate the rate ratio of COVID-19 infection in Louisiana census tracts by ADI quintile with the least deprived neighborhood as the reference. An indicator for rural-urban location was also included in the model. An offset variable was used, and the model was corrected for over dispersion.

## Results

There was a substantial difference between the ADI of the least deprived and most deprived of the 1,127 Louisiana census tracts (neighborhoods). The overall median (IQR) ADI for Louisiana was 104.32 (76.00), with the most deprived neighborhood having an ADI of 136.00, and the least deprived neighborhood having an ADI of -33.87. While the median ADI of the least deprived quintile was 76.00, the median ADI of the most deprived quintile was 118.45 (Table 1).

**Table 1:** Area Deprivation Index (ADI) Quintiles (Q) in Louisiana census tracts (N = 1127).

	LA	Q1	Q2	Q3	Q4	Q5
	(Overall)	(Least Deprived)				(Most Deprived)
Mean	**100**	69.29	96.25	104.26	110.85	119.30
Std Dev.	**20.00**	20.09	3.03	1.98	1.91	4.61
Median	**104.32**	76.00	96.64	104.32	110.94	118.48
IQR	**18.82**	20.09	5.02	3.50	3.46	5.37
Minimum	**-33.87**	-33.87	89.82	100.88	107.65	114.01
Maximum	**136.00**	89.61	100.86	107.62	114.00	136.00

Table 2 shows the median and interquartile range of census indicators that were used in the calculation of ADI. The most deprived neighborhoods in Louisiana had 31.02% of families below poverty. Similarly, more than 15.47% of occupied housing units in the most deprived neighborhoods lacked a motor vehicle. The unemployment rate was more than twice as high in the deprived neighborhoods as the less deprived neighborhoods. Almost 3% of households in the most deprived neighborhoods had more than one person per room. Similarly, the median home value in the most deprived neighborhood was substantially lower than those in the least deprived neighborhoods ($74,550 vs $273,900). These results suggest that poor people with lower levels of education were clustered together in Louisiana.

**Table 2:** Median and IQR values of census tract level indicators in Louisiana.

Indicators	Least Deprived Neighborhoods (Q1)		Most Deprived Neighborhoods (Q5)	
	Median	IQR	Median	IQR
Percent of population aged ≥ 25 years with < 9 years of education	1.71	2.29	6.68	5.97
Percent of population aged ≥ 25 years with > = to a high school	94.57	5.40	75.48	10.50
Percent of employed person ≥16 years of age in white-collar occupations	49.79	14.50	20.13	10.63
Median family income ($)	96,071	29,940	32,410	11,959
Income disparity*	0.83	0.49	1.82	0.47
Median home value ($)	273,900	112,600	74,550	21,750
Median gross rent ($)	1106	280	687	203
Median monthly mortgage ($)	1803	575	881	171
Percent of owner-occupied housing units	71.19	30.11	47.18	28.01
Percent of civilian labor force population ≥ 16 years of age unemployed	4.09	3.36	10.61	9.03
Percent of families below the poverty level	4.95	5.71	31.02	16.70
Percent of the population below 150% of the poverty threshold	14.44	12.07	53.15	15.06
Percent of single-parent households with children < 18 years of age	6.94	5.87	20.33	11.64
Percent of occupied housing units without a motor vehicle	4.11	6.53	15.47	13.78
Percent of occupied housing units without a telephone	1.50	1.60	3.25	3.15
Percent of occupied housing units without complete plumbing	0.00	0.00	0.00	0.92
Percent of occupied housing units with more than one person per room	0.62	1.48	2.87	3.88

*Income disparity was defined as the log of 100*ratio of the number of households with <$10,000 income to the number of households with $50,000+ income

From the Poisson regression analysis (Table 3) we observed that compared to the least deprived quintile of neighborhood deprivation, people living in neighborhoods with greater deprivation had a higher rate of COVID-19 infection. In the crude model (model 1) the most deprived neighborhoods (5^th^ quintile) had a 30% higher rate of COVID-19 infection compared to those in the least deprived (1^st^ quintile) neighborhoods (RR = 1.30, 95% CI = 1.17–1.39). In model 2, we adjusted for the effect of urban residence on the association between ADI and COVID-19 infection rate. Not only were the rate ratios not attenuated, they increased. In this model, those living in the most deprived neighborhood (5^th^ quintile) had a 39% higher rate of COVID-19 infection compared to those living in the least deprived neighborhood (1^st^ quintile), after adjusting for urban/rural location. Although urban location was also significantly associated with COVID-19 infection (RR 1.32, 95% CI 1.22–1.43), there was no substantial effect of urban location on the relationship between ADI quintiles and COVID-19 infection (interaction term estimate: 0.0010, p = 0.4729, data not shown).

**Table 3:** Relationship between quintiles of neighborhood deprivation as measured by the ADI and COVID-19 rates in Louisiana census tracts (N = 1127).

	Model 1*		Model 2**	
Variable	RR	95% CI	RR	95% CI
Area Deprivation Index (ADI)				
Quintile 1 (least deprived)	Ref	-	Ref	-
Quintile 2	1.06	(0.97–1.16)	1.07	(0.98–1.17)
Quintile 3	1.18	(1.1–1.28)	1.22	(1.11–1.33)
Quintile 4	1.10	(1.01–1.21)	1.19	(1.08–1.30)
Quintile 5 (most deprived)	1.30	(1.17–1.39)	1.39	(1.27–1.52)
Location (urban)			1.32	(1.22–1.43)

*Model 1 is the crude model that assesses the relationship between ADI quintiles and rates of COVID-19 infection in Louisiana.

**Model 2 is the association between ADI quintiles and rates of COVID-19 infection in Louisiana adjusted for location (rural vs urban).

Note: Quintile 1 is the reference group and refers to the least deprived neighborhoods and quintile 5 refers to the most deprived neighborhoods.

**Fig 1:**
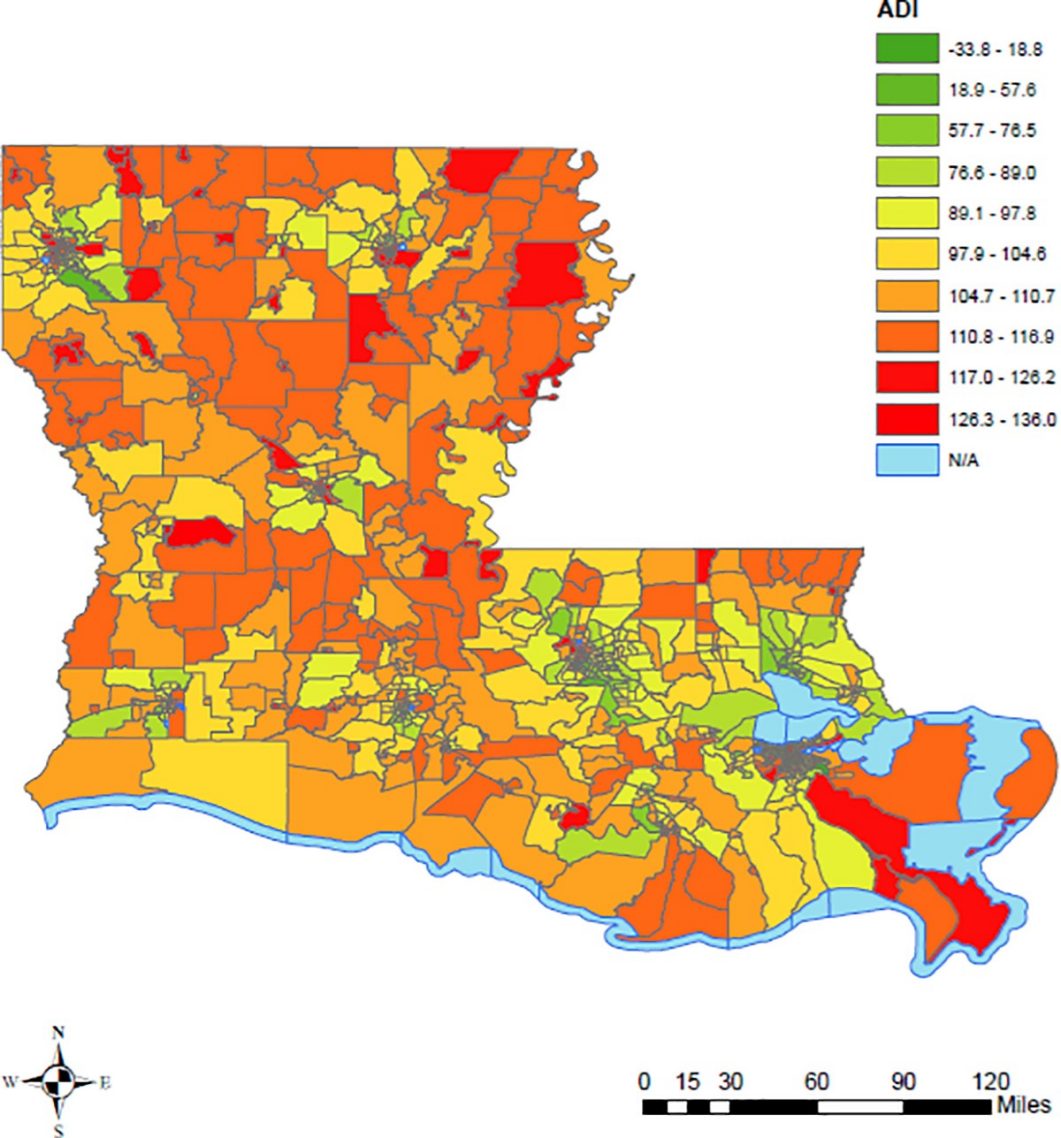
ADI in Louisiana census tracts, 2019 US census TIGER/Line shapefiles.

**Fig 2:**
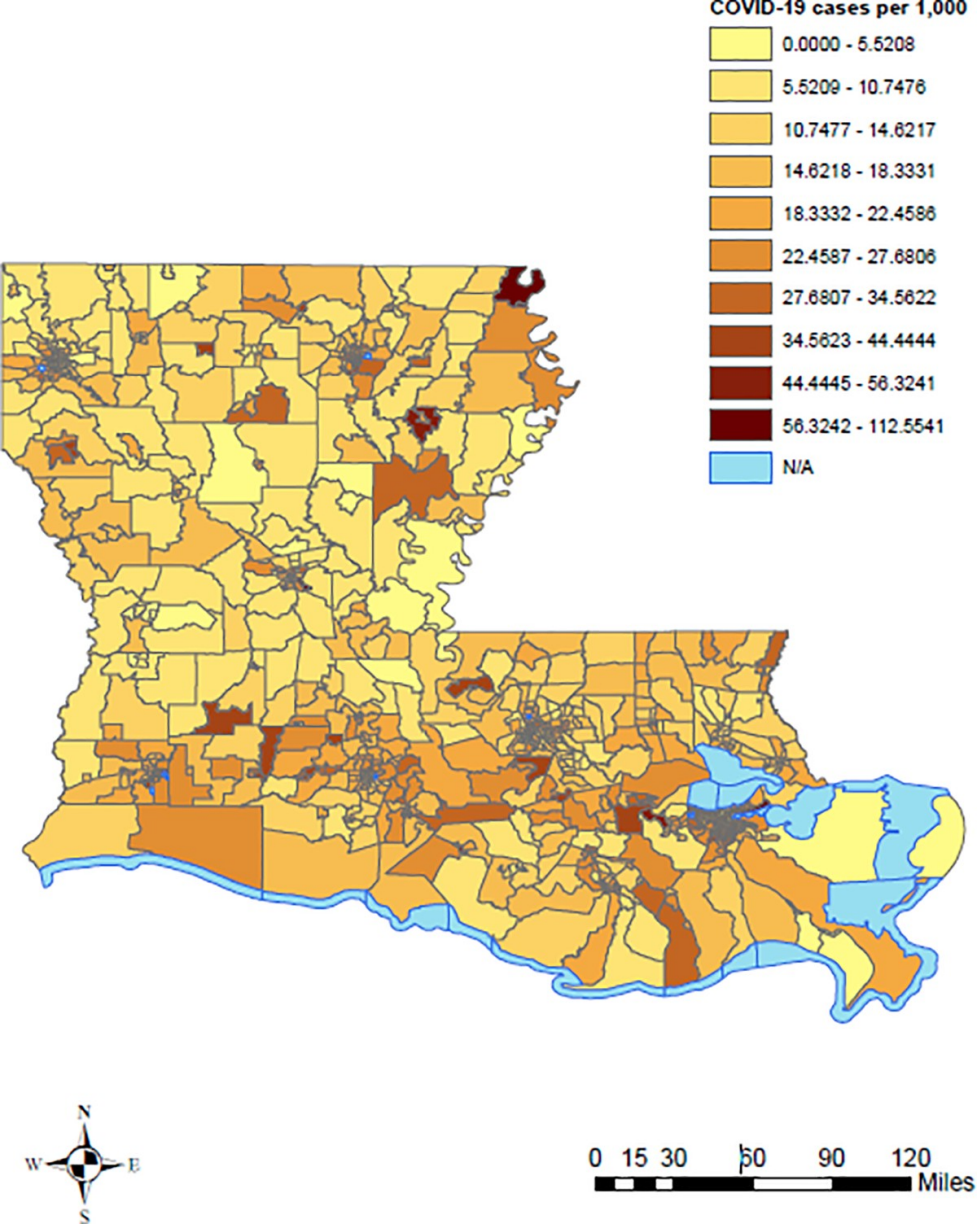
COVID-19 cases per 1,000 persons in Louisiana by census tract, 2019 US Census TIGER/Line shapefiles.

**Fig 3:**
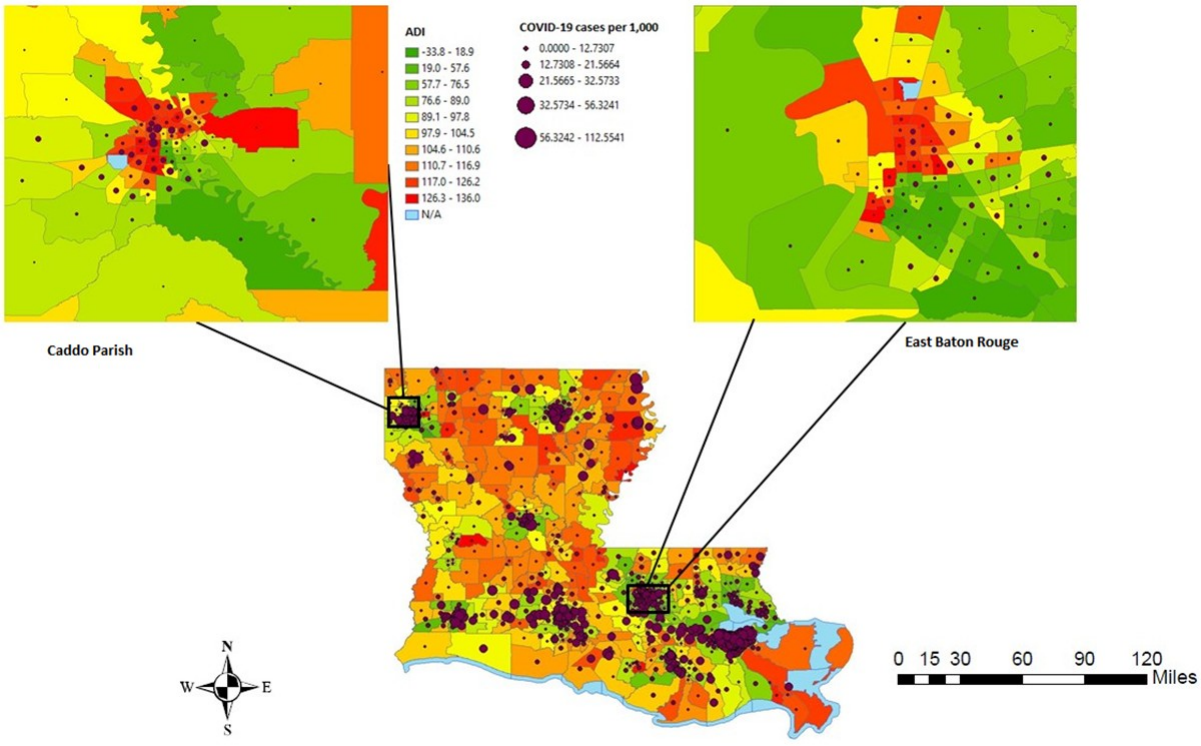
ADI and COVID-19 cases per 1,000 persons in Louisiana by census tracts, 2019 US census TIGER/Line shapefiles.

In Fig 1, the census tracts in red represent the most deprived neighborhoods, while the census tracts in green are the least deprived neighborhoods in Louisiana. In Fig 2, the census tracts in yellow represent census tracts with fewer COVID-19 cases per 1,000 persons as of July 31, 2020, while the census tracts in brown and dark brown represent higher COVID-19 cases per 1,000 persons. Fig 3 shows the distribution of ADI and COVID-19 cases per 1,000 persons simultaneously in Louisiana by census tracts.

## Discussion

To our knowledge, this is the first study to investigate the role of neighborhood deprivation on COVID-19 in Louisiana. While previous studies were limited to a very few socio-environmental factors, we used a composite area-based deprivation index to identify neighborhood deprivation in Louisiana, US. The ADI includes 17 US census indicators and could serve as an important tool in assessing the role of the neighborhood on COVID-19 disease. Our findings demonstrated the increased rate of COVID-19 cases among individuals who live in the most deprived neighborhoods compared to individuals residing in the least deprived neighborhoods.

To date, very few studies on this topic have been published. A similar study used data from seven US states (Arizona, Florida, Illinois, Maryland, North Carolina, South Carolina, and Virginia) and showed a positive correlation between COVID-19 cases and ADI [48]. Another study in New York showed higher infection rates in low-income communities in New York City compared to high-income communities [49, 50].

The neighborhood or built environment can impact health status either by influencing the available resources in the environment or by influencing behaviors that impact the transmission of COVID-19. One of the major factors that might have fueled the spread of COVID-19 disease in poor neighborhoods is likely to be overcrowded living spaces. A study conducted by Emeruwa et al. observed a strong association between neighborhood socioeconomic status and household crowding and COVID-19 cases in New York City [50]. The odds of infection were twice as high among individuals who lived in households with greater crowding (interdecile OR, 2.27 [95% CI, 1.12–4.61]). Similarly, a study in California showed 3.7 times the rate of confirmed COVID-19 cases in overcrowded neighborhoods compared to less crowded neighborhoods [51]. These findings illustrate how the housing environment plays an important role in disease dynamics and in determining the health of individuals. Neighborhood socioeconomic status and overcrowded housing may explain why non-Hispanic African American and Hispanic populations are at higher risk of getting COVID-19. Recent studies by Choi et al. and Divringi et al. have both observed an association between neighborhood characteristics (e.g. neighborhood SES) and a greater risk of COVID-19 infection among individuals residing in lower-income neighborhoods [52, 53].

A number of explanations are possible for why COVID-19 cumulative incidence in this study differed by neighborhood disadvantage as measured by ADI. Deprived neighborhoods tend to have over-crowded housing which increases the risk of transmission of COVID-19. In addition to overcrowding and neighborhood-level SES, the disparities in COVID-19 cases between neighborhoods might be directly related to the nature of residents’ occupations, a lack of telecommunication infrastructure, use of public transportation, and utility disruptions.

Another risk factor that could potentially increase an individual’s risk of contracting infection is occupation. Although many individuals have been practicing social distancing by working from home, 71% of American workers cannot work from home [54]. Individuals in certain blue-collar jobs such as construction workers, production line workers, driving tend to have a higher incidence of and mortality from COVID-19 [55]. Similarly, low-income individuals that do not have private vehicles and rely on public transportation are at higher risk of contracting COVID-19. An early study in China observed a positive association between the frequency of public transportation use and cumulative cases of COVID-19. However, the results may not be generalizable to areas where public transportation is not available, especially in rural Louisiana. In New York, Carrion et al. reported higher subway ridership among individuals who reside in neighborhoods where COVID-19 cases were higher [24]. Utility disruption is another risk factor that potentially increases the individual’s risk of contracting COVID-19. Individuals residing in the housing that do not have complete plumbing are at higher risk of COVID-19 infection. Recent studies on wastewater reported that the COVID-19 virus could remain infectious in water contaminated with feces for days to weeks [56, 57]. Symptomatic cases of COVID-19 may be easily identified and isolated to help prevent the spread of disease; however, asymptomatic cases are much less easily identified, particularly without widespread access to testing, and many symptomatic individuals do not have the ability to isolate due to lack of sick leave or because they are essential workers. Such individuals are likely to spread the disease more rapidly. As of October 12, 2020, there were approximately 200 worksite outbreaks in Louisiana [2]. The majority of studies have emphasized how adversely affected by COVID-19 certain racial and ethnic communities are; however, these groups of people may have differential exposure to the virus due to long-standing systemic health and social inequalities.

This study has several limitations. Due to a lack of data, we were unable to account for COVID-19 testing per census tract in our statistical analysis or perform a time series analysis of COVID-19 case counts. Similarly, data on COVID-19 testing was not available. In addition, this study was limited to the use of COVID-19 cases per 1,000 persons in Louisiana census tracts; data on severe outcomes, such as hospitalizations, Intensive Care Unit (ICU) admissions, and mortality were not available. Another limitation is that the impact of race could not be examined due to the lack of data by race.

A key strength of this study is the use of the ADI to characterize neighborhood disadvantage. The ADI is a validated composite index that is becoming more widely used to assess neighborhood disadvantage. The ADI provides a robust method to identify and classify deprived neighborhoods. The use of the most relevant social determinants of health in the calculation of ADI allows for better contextualization of the neighborhood.

This study contributes to the literature on social determinants of health and COVID-19 by demonstrating the impact of neighborhood deprivation on COVID-19 cases in Louisiana. Findings may help authorities to prioritize the public health response especially by increasing free testing sites and contact tracing in targeted areas. In addition, it is important to promote public health prevention measures for case isolation and quarantine of close contacts, as well as social distancing, wearing a mask while in public, and frequent handwashing to ultimately reduce the spread of COVID-19 in the most vulnerable populations. To help mitigate health disparities, policy makers could use metrics such as the ADI to target deprived neighborhoods for further resource deployment and policy decisions in response to health crises and natural disasters.

## Conclusion

We observed a great disparity in deprivation among Louisiana neighborhoods. We also found an association between neighborhood deprivation and cumulative COVID-19 cases per 1,000 persons in Louisiana. Future studies should explore specific mechanisms behind this association.

## References

[1] Louisiana Department of Health. Gov. Edwards Confirms Louisiana's First Presumptive Positive Case of COVID-19. 2020 07/06/2020.

[2] Louisiana Department of Health. Louisiana Coronavirus (COVID-19) Information In: Louisiana Department of Health, editor. COVID-19: LDH; 2020.

[3] The Wall Street Journal. The Next Coronavirus Hot Spot: Louisiana Races to Prepare for the Worst: As number of cases jump, ‘It’s like preparing for an invisible hurricane. In: Calvert S, editor.: WSJ; 2020.

[4] Office of the Governor. COVID-19 Louisiana Response Louisiana Office of the Governor, Louisiana 2020 March 23.

[5] Chowkwanyun M, Reed AL Jr., Racial Health Disparities and Covid-19—Caution and Context. N Engl J Med. 2020;383(3):201–3. 10.1056/NEJMp2012910 32374952

[6] Selden TM, Berdahl TA. COVID-19 And Racial/Ethnic Disparities In Health Risk, Employment, And Household Composition. Health Aff (Millwood). 2020:101377hlthaff202000897. 10.1377/hlthaff.2020.00897 32663045

[7] Centers for Disease Control and Prevetion. A weekly Surveillance Summary of U.S. COVID-19 Activity. Atlanta, GA CDC, National Center for Immunization and Respiratory Diseases (NCIRD) DoVD 7 10, 2020.

[8] Montenovo L, Jiang X, Rojas LP, Schmutte IM, Simon IK, Weinberg AB, et al Determinants of disparities in COVID-19 job losses. Cambridge, MA NATIONAL BUREAU OF ECONOMIC RESEARCH; 2020.

[9] Fairlie R, Xu H, Couch K. THE IMPACTS OF COVID-19 ON MINORITY UNEMPLOYMENT: FIRST EVIDENCE FROM APRIL 2020 CPS MICRODATA. Cambridge, MA NATIONAL BUREAU OF ECONOMIC RESEARCH; 2020.

[10] Wang X, Fang X, Cai Z, Wu X, Gao X, Min J, et al Comorbid Chronic Diseases and Acute Organ Injuries Are Strongly Correlated with Disease Severity and Mortality among COVID-19 Patients: A Systemic Review and Meta-Analysis. Research (Wash D C). 2020;2020:2402961 10.34133/2020/2402961 32377638 PMC7187729

[11] Liu H, Chen S, Liu M, Nie H, Lu H. Comorbid Chronic Diseases are Strongly Correlated with Disease Severity among COVID-19 Patients: A Systematic Review and Meta-Analysis. Aging Dis. 2020;11(3):668–78. 10.14336/AD.2020.0502 32489711 PMC7220287

[12] Team CC-R. Preliminary Estimates of the Prevalence of Selected Underlying Health Conditions Among Patients with Coronavirus Disease 2019—United States, February 12-March 28, 2020. MMWR Morb Mortal Wkly Rep. 2020;69(13):382–6. 10.15585/mmwr.mm6913e2 32240123 PMC7119513

[13] Partnership to fight chronic disease. WHAT IS THE IMPACT OF CHRONIC DISEASE ON LOUISIANA?: FightChronicDisease.org; 2020 2017.

[14] Gradin C. Poverty among minorities in the United States: explaining the racial poverty gap for Blacks and Latinos. Appl Econ. 2012;44(29):3793–804.

[15] Firebaugha G, Acciaia F. For blacks in America, the gap in neighborhood poverty has declined faster than segregation. P Natl Acad Sci USA. 2016;113(47):13372–7. 10.1073/pnas.1607220113 PMC5127296 27821759

[16] Wilder JM. The Disproportionate Impact of COVID-19 on Racial and Ethnic Minorities in the United States. Clin Infect Dis. 2020 10.1093/cid/ciaa959 32648581 PMC7454466

[17] Tai DBG, Shah A, Doubeni CA, Sia IG, Wieland ML. The Disproportionate Impact of COVID-19 on Racial and Ethnic Minorities in the United States. Clin Infect Dis. 2020 10.1093/cid/ciaa815 32562416 PMC7337626

[18] Koma W, Artiga S, Neuman T, Claxton G, Rae M, Kates J, et al Low-Income and Communities of Color at Higher Risk of Serious Illness if Infected with Coronavirus. KFF May 07, 2020.

[19] Millett GA, Jones AT, Benkeser D, Baral S, Mercer L, Beyrer C, et al Assessing Differential Impacts of COVID-19 on Black Communities. Ann Epidemiol. 2020 10.1016/j.annepidem.2020.05.003 32419766 PMC7224670

[20] Schneider B. COVID-19 Cases Heaviest in Poor Neighborhoods. SF Weekly.

[21] Roux AVD, Mair C. Neighborhoods and health. Ann Ny Acad Sci. 2010;1186:125–45. 10.1111/j.1749-6632.2009.05333.x 20201871

[22] Kawachi I, Berkman L. Neighborhoods and Health: Oxford University Press.

[23] Messer LC, Laraia BA, Kaufman JS, Eyster J, Holzman C, Culhane J, et al The development of a standardized neighborhood deprivation index. J Urban Health. 2006;83(6):1041–62. 10.1007/s11524-006-9094-x 17031568 PMC3261293

[24] Carrion D, Colicino E, Pedretti NF, Arfer KB, Rush J, DeFelice N, et al Assessing capacity to social distance and neighborhood-level health disparities during the COVID-19 pandemic. medRxiv. 2020 10.1101/2020.06.02.20120790 34140520 PMC8211826

[25] Bilal U, Barber S, Diez-Roux A. Spatial Inequities in COVID-19 outcomes in Three US Cities. medRxiv.

[26] Pluemper T, Neumayer E. The COVID-19 Pandemic Predominantly Hits Poor Neighborhoods, or does it? Evidence from Germany. medRxiv. 2020:2020.05.18.20105395.

[27] Borjas GJ. Demographic Determinants of Testing Incidence and COVID-19 Infections in New York City Neighborhoods. National Bureau of Economic Research; 2020.

[28] Davilla K, Abraham M, Seaberry C. Towards Health Equity in Connecticut: The Role of Social Inequality and the Impact of COVID-19. Connecticut Connecticut Health Foundation 6 2020.

[29] Palmer RC, Ismond D, Rodriquez EJ, Kaufman JS. Social Determinants of Health: Future Directions for Health Disparities Research. Am J Public Health. 2019;109(S1):S70–S1. 10.2105/AJPH.2019.304964 30699027 PMC6356128

[30] Shah GH, Shankar P, Schwind JS, Sittaramane V. The Detrimental Impact of the COVID-19 Crisis on Health Equity and Social Determinants of Health. J Public Health Manag Pract. 2020;26(4):317–9. 10.1097/PHH.0000000000001200 32433385

[31] Takian A, Kiani MM, Khanjankhani K. COVID-19 and the need to prioritize health equity and social determinants of health. Int J Public Health. 2020;65(5):521–3. 10.1007/s00038-020-01398-z 32462311 PMC8825633

[32] Robinette JW, Charles ST, Gruenewald TL. Neighborhood Socioeconomic Status and Health: A Longitudinal Analysis. J Community Health. 2017;42(5):865–71. 10.1007/s10900-017-0327-6 28315111 PMC5601026

[33] Bosma H, van de Mheen HD, Borsboom GJ, Mackenbach JP. Neighborhood socioeconomic status and all-cause mortality. Am J Epidemiol. 2001;153(4):363–71. 10.1093/aje/153.4.363 11207154

[34] Northridge ME, Sclar ED, Biswas P. Sorting out the connections between the built environment and health: a conceptual framework for navigating pathways and planning healthy cities. J Urban Health. 2003;80(4):556–68. 10.1093/jurban/jtg064 14709705 PMC3456215

[35] Nathan A, Villanueva K, Rozek J, Davern M, Gunn L, Trapp G, et al The Role of the Built Environment on Health Across the Life Course: A Call for CollaborACTION. Am J Health Promot. 2018;32(6):1460–8. 29972071 10.1177/0890117118779463a

[36] Jackson RJ. The impact of the built environment on health: an emerging field. Am J Public Health. 2003;93(9):1382–4. 10.2105/ajph.93.9.1382 12948946 PMC1447976

[37] Algren MH, Bak CK, Berg-Beckhoff G, Andersen PT. Health-Risk Behaviour in Deprived Neighbourhoods Compared with Non-Deprived Neighbourhoods: A Systematic Literature Review of Quantitative Observational Studies. PLoS One. 2015;10(10):e0139297 10.1371/journal.pone.0139297 26506251 PMC4624433

[38] Raifman MA, Raifman JR. Disparities in the Population at Risk of Severe Illness From COVID-19 by Race/Ethnicity and Income. Am J Prev Med. 2020;59(1):137–9. 10.1016/j.amepre.2020.04.003 32430225 PMC7183932

[39] Chen JT, Krieger N. Revealing the unequal burden of COVID-19 by income, race/ethnicity, and household crowding: US county vs. ZIP code analyses. Boston, MA Harvard Center for Population and Development Studies 2020. Contract No.: 1.

[40] Rader B, Scarpino S, Nande A, Hill A, Reiner R, Pigott D, et al Crowding and the epidemic intensity of COVID-19 transmission. medRxiv. 2020.

[41] Singh GK. Area deprivation and widening inequalities in US mortality, 1969–1998. Am J Public Health. 2003;93(7):1137–43. 10.2105/ajph.93.7.1137 12835199 PMC1447923

[42] Kind AJH, Buckingham WR. Making Neighborhood-Disadvantage Metrics Accessible—The Neighborhood Atlas. N Engl J Med. 2018;378(26):2456–8. 10.1056/NEJMp1802313 29949490 PMC6051533

[43] Krieger N, Chen JT, Waterman PD, Soobader MJ, Subramanian SV, Carson R. Geocoding and monitoring of US socioeconomic inequalities in mortality and cancer incidence: does the choice of area-based measure and geographic level matter?: the Public Health Disparities Geocoding Project. Am J Epidemiol. 2002;156(5):471–82. 10.1093/aje/kwf068 12196317

[44] U.S. Census Bureau. American Community Survey (ACS) 2018: American Fact Finder; [Available from: https://factfinder.census.gov/faces/nav/jsf/pages/download_center.xhtml.

[45] Kind AJ, Jencks S, Brock J, Yu M, Bartels C, Ehlenbach W, et al Neighborhood socioeconomic disadvantage and 30-day rehospitalization: a retrospective cohort study. Ann Intern Med. 2014;161(11):765–74. 10.7326/M13-2946 25437404 PMC4251560

[46] Knighton AJ, Savitz L, Belnap T, Stephenson B, VanDerslice J. Introduction of an Area Deprivation Index Measuring Patient Socioeconomic Status in an Integrated Health System: Implications for Population Health. EGEMS (Wash DC). 2016;4(3):1238 10.13063/2327-9214.1238 27683670 PMC5019337

[47] Economic Research Service. Rural-Urban Commuting Area (RUCA) Codes. Washington, DC: USDA; 2020.

[48] Hatef M, Elham MPH, Chang H-Y, Kitchen C, Weiner J, Kharrazi H. Assessing the Impact of Neighborhood Socioeconomic Characteristics on COVID-19 Prevalence Across Seven States in the United States. Frontiers in Public Health. 2020;8(554). 10.3389/fpubh.2020.571808 33072710 PMC7536340

[49] Reichberg SB, Mitra PP, Haghamad A, Ramrattan G, Crawford JM, Berry GJ, et al Rapid Emergence of SARS-CoV-2 in the Greater New York Metropolitan Area: Geolocation, Demographics, Positivity Rates, and Hospitalization for 46,793 Persons Tested by Northwell Health. Clin Infect Dis. 2020. 10.1093/cid/ciaa922 PMC7454448 32640030

[50] Emeruwa UN, Ona S, Shaman JL, Turitz A, Wright JD, Gyamfi-Bannerman C, et al Associations Between Built Environment, Neighborhood Socioeconomic Status, and SARS-CoV-2 Infection Among Pregnant Women in New York City. JAMA. 2020 10.1001/jama.2020.11370 32556085 PMC7303894

[51] CalMatters. The neighborhoods where COVID collides with overcrowded homes. California CalMatters; 2020.

[52] Choi K, Denice P. Neighborhood SES and the COVID-19 Pandemic Canada: University of Western Ontario; 2020 8 3, 2020.

[53] Divringi E, Davin R. Which Neighborhoods and Households Will Be Most Impacted by COVID-19? PA, USA: Federal Reserve Bank of Philadelphia; 2020 4 2020.

[54] U.S. Bureau of Labor Statistics. Workers who could work at home, did work at home, and were paid for work at home, by selected characteristics, averages for the period 2017–2018. MA: U.S. Bureau of Labor Statistics; 2018.

[55] Office for National Statistics. Male blue-collar workers ‘twice as likely to die from Covid-19’. London, UK: ONS 5 11, 2020.

[56] Gormley M, Aspray TJ, Kelly DA. COVID-19: mitigating transmission via wastewater plumbing systems. Lancet Glob Health. 2020;8(5):e643 10.1016/S2214-109X(20)30112-1 32213325 PMC7104291

[57] Arslan M, Xu B, Gamal El-Din M. Transmission of SARS-CoV-2 via fecal-oral and aerosols-borne routes: Environmental dynamics and implications for wastewater management in underprivileged societies. Sci Total Environ. 2020;743:140709 10.1016/j.scitotenv.2020.140709 32652357 PMC7332911

